# Effectiveness of A(H1N1)pdm09 Influenza Vaccine in Adults Recommended for Annual Influenza Vaccination

**DOI:** 10.1371/journal.pone.0066125

**Published:** 2013-06-20

**Authors:** Giedre Gefenaite, Margot Tacken, Jens Bos, Irina Stirbu-Wagner, Joke C. Korevaar, Ronald P. Stolk, Bert Wolters, Marc Bijl, Maarten J. Postma, Jan Wilschut, Kristin L. Nichol, Eelko Hak

**Affiliations:** 1 Department of Pharmacy, Unit of PharmacoEpidemiology & PharmacoEconomics (PE2), University of Groningen, Groningen, The Netherlands; 2 Department of Epidemiology, University Medical Centre Groningen, University of Groningen, The Netherlands; 3 Scientific Institute for Quality of Healthcare (IQ healthcare), Radboud University Nijmegen Medical Centre, Nijmegen, The Netherlands; 4 NIVEL, Netherlands Institute for Health Services Research, Utrecht, The Netherlands; 5 Community Health Services, Groningen, The Netherlands; 6 Department of Internal Medicine and Rheumatology, Martini Hospital, Groningen, The Netherlands; 7 Department of Medical Microbiology, Molecular Virology Section, University Medical Centre Groningen, University of Groningen, Groningen, The Netherlands; 8 Research Service, Veterans Affairs Medical Centre, Minneapolis, Minnesota, United States of America; 9 Department of Medicine, University of Minnesota, Minneapolis, Minnesota, United States of America; University of Hong Kong, Hong Kong

## Abstract

**Introduction:**

Because of variability in published A(H1N1)pdm09 influenza vaccine effectiveness estimates, we conducted a study in the adults belonging to the risk groups to assess the A(H1N1)pdm09 MF59-adjuvanted influenza vaccine effectiveness.

**Methods:**

VE against influenza and/or pneumonia was assessed in the cohort study (n>25000), and vaccine effectiveness against laboratory-confirmed A(H1N1)pdm09 influenza was assessed in a matched case-control study (16 pairs). Odds ratios (OR) and their 95% confidence intervals (95% CI) were calculated by using multivariate logistic regression; vaccine effectiveness was estimated as (1-odds ratio)*100%.

**Results:**

Vaccine effectiveness against laboratory-confirmed A(H1N1)pdm09 influenza and influenza and/or pneumonia was 98% (84–100%) and 33% (2–54%) respectively. The vaccine did not prevent influenza and/or pneumonia in 18–59 years old subjects, and was 49% (16–69%) effective in 60 years and older subjects.

**Conclusions:**

Even though we cannot entirely rule out that selection bias, residual confounding and/or cross-protection has played a role, the present results indicate that the MF59-adjuvanted A(H1N1)pdm09 influenza vaccine has been effective in preventing laboratory-confirmed A(H1N1)pdm09 influenza and influenza and/or pneumonia, the latter notably in 60 years and older subjects.

## Introduction

**Figure 1:**
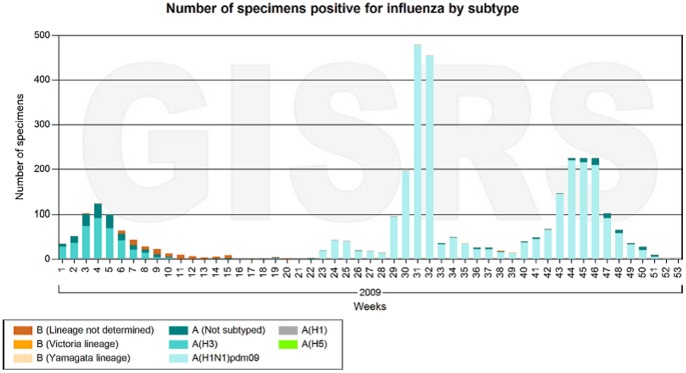
Number of specimens positive for influenza by subtype in the Netherlands [3]. Different colours mark different influenza subtypes.

Approximately six months after the emergence of A(H1N1)pdm09 influenza in April 2009, new vaccines against the pandemic A(H1N1)pdm09 virus appeared on the market. According to the Dutch Health Council advice several risk groups were recommended to receive the pandemic vaccine (see Table 1). In the Netherlands, the vaccination campaign against the influenza A(H1N1)pdm09 started at the beginning of November 2009, when the incidence of A(H1N1)pdm09 influenza reached its peak [1], [2], see Figure 1 [3]. Two doses - at least two weeks apart - were scheduled, with the influenza A(H1N1)pdm09 vaccination starting two weeks after the seasonal influenza vaccination.

**Table 1:** Organization of 2009/2010 influenza vaccination campaign in the Netherlands (based on [19], [29], [30].

Risk groups	Provider of thevaccinations	A(H1N1)pdm09vaccine used	Eligible for 2009/2010seasonal influenza vaccination
Family members and caretakers of individuals withhigh risk for severe disease or death, institutionalized individuals, those suffering from underlying medical conditions (pulmonary disease, cardiac disease,diabetes mellitus, chronic kidney failure, cancer, immunocompromising conditions), and healthyelderly > = 60 years old	General practice	Focetria^b^	Eligible
Children aged 6 months up to and including 4 yearsand household members of children youngerthan 6 months	Community Health Services	Pandemrix^a^	Not eligible
Pregnant women in their second and third trimester	General practice	Focetria^b^	Not eligible
Health care workers in contact with high risk groups	Occupational Health Physician	Focetria^b^	Eligible

a Focetria is MF59-adjuvanted vaccine produced by Novartis.

b Pandemrix is AS03-adjuvanted vaccine produced by GSK.

Studies have shown that A(H1N1)pdm09 pandemic vaccines were very immunogenic [4], [5]. However, clinical effectiveness estimates of A(H1N1)pdm09 vaccine seem to vary. In a multicentre European study the effectiveness of monovalent A(H1N1)pdm09 vaccines in preventing laboratory-confirmed influenza of about 70% was estimated [6]. The non-adjuvanted A(H1N1)pdm09 influenza vaccine appeared to be more effective in younger adults and was estimated to prevent 50 to 90% of laboratory-confirmed influenza [7], [8]. More data are available about MF59- and AS03-adjuvanted vaccines that in Europe were more commonly used during the pandemic. The AS03-adjuvanted vaccine prevented up to 95% of laboratory-confirmed influenza in the general population [9], and was much less effective (VE 41%, 95% CI −71%–80%) in the 50 years old and older risk group subjects [10]. MF59-adjuvanted vaccine effectiveness in preventing medically attended influenza-like illness (ILI) in subjects of 60 years and older was 25% [11], and it showed to be quite low against more specific laboratory-confirmed A(H1N1)pdm09 influenza outcomes as well [2], [12].

Because of the substantial variability in published vaccine effectiveness estimates and limited information on the MF59-adjuvated A(H1N1)pdm09 pandemic vaccine effectiveness in preventing different outcomes, we aimed to provide further evidence about its effectiveness against influenza and/or pneumonia, and laboratory-confirmed A(H1N1)pdm09 influenza. We achieved this by conducting a cohort and a matched case-control study in a population of adults with underlying medical conditions and (healthy) 60 years and older persons that received MF59-adjuvanted Focetria vaccine.

## Methods

### Ethics Statement

Both studies were conducted in accordance with the Dutch Law for the Protection of Personal Data (Wet Bescherming Persoonsgegevens) [13] and the Declaration of Helsinki [14]. In the case-control study, the cases agreed to participate in the study by signing and returning the informed consent and patient information forms. Based on the Dutch Law for the Protection of Personal Data no medical ethical committee approval was required for these studies, therefore the medical ethical committee was not contacted.

### The Cohort Study

We conducted a retrospective cohort study in 25743 18 years and older subjects, who belonged to one of the risk groups that were recommended to receive influenza vaccinations in 2009, i.e. adults with underlying medical conditions or 60 years and older subjects. The anonymous data were received from the Netherlands Information Network of General Practice (LINH) database, which includes demographic and clinical information collected from a representative network of general practices spread throughout the Netherlands [15]. Individuals in our cohort were vaccinated with MF59-adjuvanted pandemic influenza vaccine (Focetria® by Novartis [16]). A subject was considered as vaccinated if at least one dose of the vaccine against A(H1N1)pdm09 influenza was administered. As individual vaccination dates were not available, and the vaccination campaign took off at the beginning of November, we assumed that the vaccine would start demonstrating its effectiveness in the middle of November, i.e. > = 7 days after receiving the vaccine.

The outcome of the study was influenza and/or pneumonia coded as R80 and R81 according to the International Classification of Primary Care coding system (ICPC) [17]. The main outcome occurrence period was between November 15 and December 31, 2009. Subjects with a record of influenza after the World Health Organization declared the A(H1N1)pdm09 pandemic on June 11, 2009 and before the vaccination campaign was anticipated to have an effect (November 14, 2009) were excluded.

Several demographic and clinical characteristics were included as covariates. Demographic characteristics recorded were sex and age. Clinical characteristics included underlying medical conditions, seasonal influenza vaccination status, and a number of visits to the general practice (GP visits). Underlying medical conditions were grouped into lung diseases, diabetes mellitus, breathing problems due to neurological disorders, cardiovascular diseases, chronic kidney failure, HIV and other immunocompromising conditions (Appendix S1). Seasonal influenza vaccination was recorded positively when a single dose of a seasonal influenza vaccine in 2009 was received. A number of GP visits between October 1 in 2008 and October 1 in 2009 divided into three categories (< = 2; 3–13, and > = 14 visits) was obtained as an indicator for the severity of health problems.

To be able to address the unmeasured confounding as well as calendar time, influenza and/or pneumonia were recorded during the reference period, i.e. before the vaccine was anticipated to demonstrate its effectiveness (June 11– November 14, 2009). Odds ratio of one indicating no effectiveness of an intervention was expected, a departure from one indicating the bias.

We used descriptive statistics and univariate logistic regression to compare the characteristics of vaccinated and unvaccinated subjects. Odds ratios (OR) and their 95% confidence intervals (95% CI) derived from the univariate and multivariate logistic regression were used to estimate the unadjusted and adjusted A(H1N1)pdm09 influenza vaccine effectiveness (VE) calculated as (1-OR)*100%. The analysis was also stratified by age into the groups of 18–59 and > = 60 years old. To adjust for unmeasured confounding, we used a method proposed by Weiner et al. [18], where the odds ratio when the vaccine was expected to demonstrate an effect was divided by the odds ratio when the vaccine was expected not to demonstrate any effect. Confidence intervals were obtained by bootstrapping the original sample for 1000 times and calculating the unmeasured confounding adjusted estimates for each sample. The estimates were sorted, and the 2.5% and 97.5% quintiles indicated the lower and the upper limit of the 95% CI.

### The Matched Case-control Study

The data about the A(H1N1)pdm09 positive cases came from four Community Health Services (CHSs) who were registering laboratory-confirmed A(H1N1)pdm09 influenza cases in four provinces in the Netherlands. The patients presenting with the influenza-like illness symptoms to the GP throughout the pandemic where swabbed independent of the influenza vaccination status and when positive for A(H1N1)pdm09, reported to the CHS covering the region. The cases were matched with the controls from a previously described cohort in a ratio of 1∶10 on sex, age (number of years) and underlying medical conditions (none versus one or more).

A control subject was considered as vaccinated if at least one dose of the vaccine against A(H1N1)pdm09 influenza was recorded in the LINH database. A case was considered as vaccinated if at least one dose of A(H1N1)pdm09 vaccine was administered > = 7 days before a subject was registered with a laboratory-confirmed A(H1N1)pdm09 influenza at one of the CHSs. The main source of information of the cases was the GP files; self-reported vaccination status was used if vaccination status was not recorded in the GP files. When vaccination dates were unknown, vaccine effectiveness was calculated under the assumption that these subjects were not vaccinated and under the assumption that these subjects were vaccinated.

The main study outcome was notification with laboratory-confirmed influenza A(H1N1)pdm09 between 12 November and 31 December in 2009 (Appendix S2). The sample of controls consisted of subjects from the LINH database who were not registered with influenza during the A(H1N1)pdm09 influenza season from 11 June to 31 December in 2009.

Demographic characteristics for the cases were obtained through a questionnaire. Information about the underlying medical conditions (Appendix S3) prior to 1 October 2009 was collected from the GP files. Information about the demographic characteristics and underlying medical conditions for the controls was obtained from the LINH database.

Vaccine effectiveness (VE) was calculated by using OR and their 95% CI derived from the conditional logistic regression: VE = (1-OR)*100%.

## Results

### The Cohort Study

In total, the cohort consisted of 25743 individuals who were recommended to receive influenza vaccinations in 2009. The number of subjects included in the vaccine effectiveness analysis during the period when vaccine was anticipated to demonstrate the effect was 25568, as 175 subjects were registered with influenza before the A(H1N1)pdm09 vaccination campaign was anticipated to have an effect.

Seventy-three percent of the study population received at least one dose and 66% received two doses of A(H1N1)pdm09 influenza vaccine. Vaccinated individuals were slightly older and had more underlying medical conditions (Table 2). With respect to demographic characteristics, A(H1N1)pdm09 and seasonal influenza vaccination status, and underlying medical conditions our sample was similar to the LINH sample [19].

**Table 2:** Descriptive characteristics of subjects vaccinated and unvaccinated against A(H1N1)pdm09 influenza (N = 25568)**.**

	Vaccinated	Unvaccinated	OR (95% CI)
	N = 18774 (73.4%)	N = 6794 (26.6%)	
**Male sex**	8692 (46.3)	3316 (48.8)	0.90 (0.86–0.96)
**Age in years**	63.5 (15.1)	57.7 (16.4)	1.02 (1.02–1.03)
**Lung diseases**	4895 (26.1)	1417 (20.9)	1.34 (1.25–1.43)
**Immunocompromised conditions and HIV**	976 (5.2)	214 (3.1)	1.69 (1.45–1.96)
**Diabetes mellitus**	3535 (18.8)	726 (10.7)	1.94 (1.78–2.11)
**Cardiovascular disease**	9311 (49.6)	2103 (31.0)	2.20 (2.07–2.33)
**Kidney insufficiency**	312 (1.7)	68 (1.0)	1.67 (1.28–2.18)
**Breathing problems due to neurological disorders**	195 (1.0)	61 (0.9)	1.16 (0.87–1.55)
**GP visits: < = 2**	2806 (14.9)	2051 (30.2)	Reference
**3–13**	8983 (47.8)	3449 (50.8)	1.90 (1.78–2.04)
**> = 14**	6985 (37.2)	1294 (19.0)	3.95 (3.63–4.28)
**Seasonal influenza vaccination**	16642 (88.6)	1781 (26.2)	21.97 (20.48–23.57)

After adjustment for demographic and clinical characteristics, the odds ratios in the total sample decreased from 0.83 to 0.67, resulting in the vaccine effectiveness estimate of 33% (Table 3). The vaccine effectiveness dropped to 25% after adjusting for unmeasured confounding (0.83/1.10), although it became not statistically significant (0.75, 95% CI 0.47–1.17).

**Table 3:** A(H1N1)pdm09 influenza vaccine effectiveness to prevent influenza and/or pneumonia: a cohort study between 11 June –31 December 2009.

	Study period (November 15– December 31, 2009)				Reference period (June 11– November 14, 2009)
	Exposed	Unexposed	OR (95% CI)*	OR (95% CI)**	OR (95% CI)*
***Total sample***	*18774*	*6794*			
**Influenza and/or pneumonia**	96	42	0.83 (0.57–1.19)	0.67 (0.46–0.98)	1.10 (0.86–1.41)
***Subjects > = 60 years old***	*13410*	*3967*			
**Influenza and/or pneumonia**	52	24	0.64 (0.39–1.04)	0.51 (0.31–0.84)	1.00 (0.71–1.41)
***Subjects 18–59 years old***	*5364*	*2827*			
**Influenza and/or pneumonia**	44	18	1.29 (0.74–2.24	1.07 (0.61–1.88	1.41 (0.98–2.02

OR – odds ratio; 95% CI –95% confidence interval.

* Unadjusted.

** Adjusted for sex, age, underlying medical conditions and GP visits (stratified by age analysis adjusted only for GP visits).

When we stratified the analysis by age, the vaccine remained effective only in 60 years old and older subjects, and there seemed to be no unmeasured confounding (OR of 1 during the reference period).

### The Matched Case-control Study

Of the 119 approached individuals who were notified by the 4 GGDs with A(H1N1)pm09 influenza between 12 November and 31 December in 2009, 29 subjects (24%) decided to join the study. Sixteen subjects were eligible to be included in the final analysis because they belonged to one of the risk groups that were recommended to receive annual influenza vaccinations, i.e. had at least one underlying medical condition or were healthy 60 years or older subjects.

A dataset consisted of 16 case-control pairs matched 1∶10. The majority of cases (11/16) were subjects of 18–59 years old and had at least one underlying medical condition. Overall, the most common underlying medical conditions among the cases were lung and cardiovascular diseases, 7 and 5 respectively.

At least one dose of the A(H1N1)pdm09 influenza vaccine was received by 75% of the controls, which was similar as in the risk groups in the LINH cohort [19]. Only one subject (6.3%) received one dose of the A(H1N1)pdm09 vaccine > = 7 days before being notified with A(H1N1)pdm09 influenza, and for five cases the information about the vaccination dates was not available. When we assumed that the persons with unknown vaccination dates were vaccinated, vaccine effectiveness was 80% (41–93%), and it increased to 98% (84–100%) when we assumed that these persons were not vaccinated (Table 4).

**Table 4:** A(H1N1)pdm09 influenza vaccine effectiveness to prevent laboratory confirmed A(H1N1)pdm09 influenza: a matched case-control study between 12 November –31 December 2009.

Vaccinated cases/cases	Vaccinated controls/controls	Crude odds ratio (95% CI)	Vaccine effectiveness (95% CI)
1/16	120/160	0.02 (0.003–0.18)*	98% (82–100%)*
6/16	120/160	0.20 (0.07–0.59)**	80% (41–93%)**

* Crude odds ratio, its 95% confidence interval and the corresponding vaccine effectiveness estimates for the assumption that persons whose vaccination status was missing or unavailable were not vaccinated;

** Crude odds ratio, its 95% confidence interval and the corresponding vaccine effectiveness estimates for the assumption that persons whose vaccination status was missing or unavailable were vaccinated.

## Discussion

Our results indicate that immunization with MF59-adjuvanted vaccine was preventive against laboratory-confirmed A(H1N1)pdm09 influenza. Furthermore, vaccine effectiveness against influenza and/or pneumonia increased after adjusting for measured confounding, although the estimate was not statistically significant after adjusting for unmeasured confounding. The results of the cohort study have also suggested that the vaccine was only effective in 60 years old and older individuals, and in this group there seemed to be no unmeasured confounding. Higher vaccine effectiveness in this group might be explained by cross-protective antibodies due to previous exposure to H1N1 influenza [20] as subjects born before 1950 and who were probably exposed to a descendant of the 1918 H1N1 pandemic virus had higher antibody titres against A(H1N1)pdm09 influenza than younger individuals [21], [22].

### Strengths and Limitations

In our study we were able to assess both, more and less specific outcomes such as laboratory-confirmed influenza and influenza and/or pneumonia as registered in the GP database. Nevertheless, some selection bias might have occurred in our outcome measure since influenza is not clearly distinguishable from other acute respiratory infections on the basis of its clinical profile, and therefore influenza cases in our cohort might represent influenza-like illness rather than influenza. On the other hand, as the main circulating virus in 2009/2010 season was A(H1N1)pdm09, patients presenting themselves with influenza-like symptoms were most likely registered as influenza. Some potential for biases in a case-control study might have occurred because of different sources of data on cases and controls as well: only the individuals that did not have registered visits due to influenza (R80 code according to ICPC) during the A(H1N1)pdm09 influenza pandemic between 11 June –31 December, 2009 were included as controls, while cases were subjects with laboratory-confirmed A(H1N1)pdm09 influenza throughout the study period. As virological testing was only done in the general practice for severe influenza cases, patients with less severe influenza who did not contact the GP were not captured. Hence, A(H1N1)pdm09 influenza incidence in the cohort, and therefore control population of a case-control study, might be underestimated and therefore lead to vaccine effectiveness underestimation.

Although A(H1N1)pdm09 influenza vaccination status was recorded, the individual vaccination dates of the cohort were not known. We therefore limited the period when we assessed the vaccine effectiveness to when we anticipated that the vaccine would already have had an effect. When the vaccination dates of cases were not known, we performed the statistical analysis under two scenarios, when we assumed that the subjects with unknown vaccination dates were vaccinated, and when we assumed that the subjects with unknown vaccination dates were not vaccinated. However, due to a moderate response rate of the cases and absence of information on vaccination status for non-responders, we cannot exclude the self-selection bias due to vaccination status of the responders. Still, since the estimated vaccine effectiveness was reaching more than 80%, it is unlikely that such selection bias can explain the findings.

As evidence about influenza vaccine effectiveness usually comes from observational studies, efforts should be put to control for bias and confounding. When the nature of the data allows it, adjustment for measured as well as unmeasured confounding should therefore be incorporated [23], [24]. Although we were not able to address the unmeasured confounding in our case-control study, by matching on several baseline characteristics, we addressed some of the measured confounding. In our cohort study we adjusted for measured confounding and we controlled for unmeasured confounding by dividing the vaccine effectiveness estimates during the period when we anticipated the vaccine to have an effect by an estimate during a reference period when we did not anticipate the vaccine to have an effect.

Use of diagnoses of underlying medical conditions based on ICPC, although might have some coding errors and disease severity measure is not directly available, is otherwise a valid and reliable way to measure health in large populations [25], [26]. Use of GP based data also reassures continuity of the data. Besides, only the records from the GPs that delivered good-quality data to LINH were made available for this study.

Finally, our results should not be directly extrapolated to the situation of seasonal influenza because the affected vulnerable groups during the seasonal and the A(H1N1)pdm09 influenza seasons appeared to differ. The population severely affected during the 2009 pandemic was younger as compared to the population usually affected by the seasonal influenza [27]. As compared to the seasonal virus H3N2, A(H1N1)pdm09 influenza was also less severe in terms of significant cause of illness in older adults [28].

Additionally, seasonal and pandemic vaccination statuses were highly correlated as nearly the same population received both vaccines. To avoid multicolinearity we therefore did not include seasonal influenza vaccination status as a covariate in the multiple logistic regression model. Moreover, as there is not enough evidence about the cross-protection by the seasonal influenza vaccine against the A(H1N1)pdm09, we did not assess seasonal influenza vaccine effectiveness against the A(H1N1)pdm09 influenza.

### Conclusion

The vaccine against A(H1N1)pdm09 influenza appeared to be effective against laboratory-confirmed influenza. The vaccine was also effective against influenza and/or pneumonia, notably in subjects of 60 years and older. It is difficult to estimate what (if any) part of the effectiveness of the vaccine in 60 years and older subjects might be explained by previous cross-protection.

## Supporting Information

Appendix S1 **Definitions of underlying medical conditions in cases.**
(DOC)

Appendix S2 **Notification of laboratory confirmed A(H1N1)pdm09 dates of cases from a matched case-control study.**
(DOC)

Appendix S3 **Definitions of underlying medical conditions in controls.**
(DOC)
