# Supplementary material for: Effectiveness of A(H1N1)pdm09 Influenza Vaccine in Adults Recommended for Annual Influenza Vaccination
Source: PLoS One. 2013 Jun 20;8(6):e66125. doi: 10.1371/journal.pone.0066125 (PMC3688717; doi:10.1371/journal.pone.0066125)
Supplement: Appendix S1 — Definitions of underlying medical conditions in cases. (DOC) [file pone.0066125.s001.doc]

**Appendix S**1. Definitions of underlying medical conditions in cases.

| **Underlying medical conditions** | **ICPC* codes** |
| --- | --- |
| Cardiovascular conditions | K74 K78 K77 K80.03 K82 K83 K84 |
| Lung diseases | R84 R85 R91 R95 |
| Diabetes Mellitus | T90 |
| Chronic kidney insufficiency | U85 U88 U99 |
| Immunocompromising conditions | T99.08 T99.09 T99.10 D97 |
| HIV | B90 |
| Breathing problems due to neurological disorders | N86 N87 N99 |

*International Classification of Primary Care (ICPC) coding system.
