# Supplementary material for: Effectiveness of A(H1N1)pdm09 Influenza Vaccine in Adults Recommended for Annual Influenza Vaccination
Source: PLoS One. 2013 Jun 20;8(6):e66125. doi: 10.1371/journal.pone.0066125 (PMC3688717; doi:10.1371/journal.pone.0066125)
Supplement: Appendix S2 — Notification of laboratory confirmed A(H1N1)pdm09 dates of cases from a matched case-control study. (DOC) [file pone.0066125.s002.doc]

**Appendix S2. Notification of laboratory confirmed A(H1N1)pdm09 dates of cases from a matched case-control study.**

| **Number of cases** | **Date of A(H1N1)pmd09 notification** |
| --- | --- |
| 1 | 12-11-2009 |
| 1 | 14-11-2009 |
| 3 | 15-11-2009 |
| 1 | 16-11-2009 |
| 2 | 17-11-2009 |
| 3 | 18-11-2009 |
| 1 | 24-11-2009 |
| 1 | 01-12-2009 |
| 1 | 04-12-2009 |
| 1 | 14-12-2009 |
| 1 | 21-12-2009 |
| Total number of cases: 16 |  |
