# Supplementary material for: Effectiveness of A(H1N1)pdm09 Influenza Vaccine in Adults Recommended for Annual Influenza Vaccination
Source: PLoS One. 2013 Jun 20;8(6):e66125. doi: 10.1371/journal.pone.0066125 (PMC3688717; doi:10.1371/journal.pone.0066125)
Supplement: Appendix S3 — Definitions of underlying medical conditions in controls. (DOC) [file pone.0066125.s003.doc]

**Appendix S3**. Definitions of underlying medical conditions in controls.

| **Chronic underlying conditions** | ***ICPC codes** | ****ATC codes** | | |
| --- | --- | --- | --- | --- |
| **Cardiovascular conditions** | K71 | A09AA02 | C01DA02 | C07AB08 |
|  | K73 | A12CC01 | C01DA08 | C07AB09 |
|  | K74 | A12CC02 | C01DA14 | C07AB12 |
|  | K75 | B01AB04 | C01DX16 | C07AG01 |
|  | K76 | B01AB05 | C01EB10 | C07AG02 |
|  | K77 | B01AB06 | C01EB17 | C08CA01 |
|  | K78 | B01AC04 | C02CA01 | C08CA02 |
|  | K80 (except for K80.03) | B01AC06 | C02DB02 | C08CA05 |
|  | K80.03 | B01AC08 | C03AA03 | C08DA01 |
|  | K82 | B01AC13 | C03AA04 | C08DA01 |
|  | K83 | B01AC16 | C03BA04 | C08DB01 |
|  | K84 | B01AC17 | C03CA01 | C09AA |
|  | K84 | C01AA05 | C03CA02 | C09AA01 |
|  | K90 | C01BA01 | C03DA01 | C09AA02 |
|  |  | C01BA02 | C03DA04 | C09AA03 |
|  |  | C01BA03 | C03DB02 | C09AA04 |
|  |  | C01BB04 | C03EA03 | C09AA05 |
|  |  | C01BC03 | C07AA02 | C09AA06 |
|  |  | C01BC04 | C07AA03 | C09AA07 |
|  |  | C01BD01 | C07AA05 | C09AA08 |
|  |  | C01BD05 | C07AA07 | C09AA09 |
|  |  | C01CA07 | C07AB02 | C09AA10 |
|  |  | C01CA16 | C07AB03 | C09CA03 |
|  |  | C01CE02 | C07AB04 | C09CA06 |
|  |  | C01CE03 | C07AB05 | N03AB02 |
|  |  | C01CX08 | C07AB07 | S01ED02 |
| **Lung diseases** | K93 | A10AC01 | A10BH02 | R03AK06 |
|  | L82 | A10AD04 | A10BX02 | R03AK07 |
|  | L85 | A10AD05 | A10BX03 | R03BA01 |
|  | R70 | A10AE01 | A10BX04 | R03BA01 |
|  | R82 | A10AE04 | H02AA02 | R03BA01 |
|  | R84 | A10AE05 | H02AB10 | R03BA01 |
|  | R85 | A10BA02 | J01GB01 | R03BA01 |
|  | R86 | A10BB01 | J01MA12 | R03BA01 |
|  | R89 | A10BB03 | J01MA14 | R03BA02 |
|  | R91 | A10BB09 | L01XE02 | R03BA05 |
|  | R95 | A10BB12 | L01XE02 | R03BB01 |
|  | R96 | A10BF01 | H02AB09 | R03BB04 |
|  | R99 | A10BF02 | P01CX01 | R03CC02 |
|  |  | A10BF03 | R01AD08 | R03CC03 |
|  |  | A10BG02 | R03AC02 | R03DA04 |
|  |  | A10BG03 | R03AC03 | R05CB01 |
|  |  | A10BH01 | R03AC12 | R05CB13 |
|  |  |  | R03AC13 | V03AB23 |
|  |  |  | R03AK04 | A10AB04 |
| **Diabetes Mellitus** | T90 | A10AB01 |  |  |
| **Chronic kidney insufficiency** | U85 | H02AB04 |  |  |
|  | U88 |  |  |  |
|  | U99 |  |  |  |
| **Immunocompromising conditions** | B72 | A09AA02 | L01CA02 | L01XC07 |
|  | B73 | A12CC01 | L01CA04 | L01XD01 |
|  | B74 | J01FA09 | L01CB01 | L01XX17 |
|  | B76 | J01FA10 | L01AA06 | L01XX34 |
|  | D97 | L01AA01 | L01CD01 | L02BG01 |
|  | T99 (except T.99.08, T99.09 and T99.10) | L01AA05 | L01CD02 | N01BB02 |
|  | T99.08 | L01AD02 | L01DB01 | N02AA01 |
|  | T99.09 | L01BA04 | L01XA01 |  |
|  | T99.10 |  |  |  |
| **HIV** | B90 | J05AE01 | J05AF02 | J05AG03 |
|  |  | J05AE02 | J05AF03 | J05AR01 |
|  |  | J05AE03 | J05AF04 | J05AR02 |
|  |  | J05AE04 | J05AE02 | J05AR03 |
|  |  | J05AE05 | J05AF05 | J05AR04 |
|  |  | J05AE06 | J05AF07 | J05AR06 |
|  |  | J05AE08 | J05AF06 | J05AX07 |
|  |  | J05AE09 | J05AF09 | J05AX08 |
|  |  | J05AE10 | J05AG01 | J05AX09 |
|  |  | J05AF01 |  |  |
| **Breathing problems due to neurological disorders** | N86 |  |  |  |
|  | N87 |  |  |  |
|  | N99 |  |  |  |

The table is adapted from “Monitoring vaccination coverage of National Influenza Prevention programme” (in Dutch “Monitoring vaccinatiegraad Nationaal Programma Grieppreventie 2009”) [19].

*International Classification of Primary Care (ICPC) coding system;

**Anatomical Therapeutic Chemical (ATC) classification system.
